# Colorimetric and Smartphone-Based Dual-Mode Rapid Detection of Congo Red Using Iron Oxide Quantum Dots

**DOI:** 10.1021/acsomega.4c08644

**Published:** 2024-11-07

**Authors:** Sri Sudewi, Chien-Hung Li, Venkata Sai Sashankh Penki, Muhammad Zulfajri, Naorem Jemes Meitei, Genin Gary Huang

**Affiliations:** † Department of Pharmacy, Faculty of Mathematics and Natural Science, Universitas Sam Ratulangi, Manado 95115, Indonesia; ‡ Department of Medicinal and Applied Chemistry, Kaohsiung Medical University, Kaohsiung 80708, Taiwan; § Department of Chemistry Education, Universitas Serambi Mekkah, Banda Aceh, Aceh 23245, Indonesia; ∥ Department of Medical Research, Kaohsiung Medical University Hospital, Kaohsiung 80708, Taiwan; ⊥ Department of Chemistry, National Sun Yat-Sen University, Kaohsiung 80424, Taiwan

## Abstract

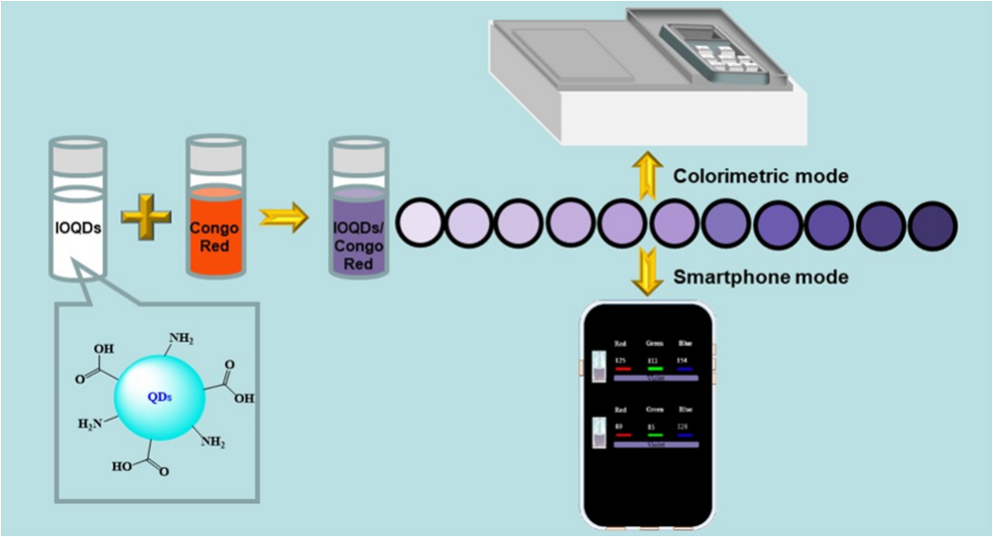

Congo red is toxic to humans and the environment and persists in the environment for long periods. Therefore, developing a rapid detection method for Congo red is crucial. In this study, iron oxide quantum dots (IOQDs) were synthesized and employed for dual-mode detection (colorimetric and smartphone-based) of Congo red in real samples. Upon mixing with Congo red, the IOQDs induce a color change in the solution due to the strong intermolecular interactions between Congo red and the IOQDs, making them practical as colorimetric sensors. To further increase the on-site detection capabilities of IOQDs, images of the sensor platform were captured using a smartphone, and the color data were analyzed with a dedicated APP. As a colorimetric sensor, the ultraviolet–visible (UV–vis) absorbance response exhibited good linearity for Congo red concentrations between 2 and 50 μM, with a detection limit of 0.89 μM. The smartphone-based sensor also provided highly quantitative results, showing a linear relationship between Congo red concentrations and the blue-to-red (B/R) channel ratio, with a detection limit of 0.58 μM. Moreover, this dual-mode method demonstrated better selectivity for Congo red than other colorimetric sensors, even in the presence of other red dyes. The proposed method is convenient, fast, low-cost, and suitable for real-sample applications.

## Introduction

More and more research has recently been reported on developing colorimetric nanosensors utilizing quantum dots (QDs) for on-site sensing with visual detection.^1−3^ One of the ways to image analytes with QDs is the ratiometric fluorescence approach.^3^ For on-site visualized copper ion measurements, Yao’s group built a ratiometric fluorescence nanohybrid probe using two different-sized cadmium telluride QDs that emit red and green fluorescence.^3^ The second method used a hybrid of plasmonic gold nanoparticles (NPs) and carbon QDs, which serve as a dual-mode colorimetric and fluorometric nanosensor for detecting glutathione.^4^ Another way fluorescence and colorimetric sensors are reported involves inducing the aggregation of the QDs as they interact with analytes. This mechanism alters the photoluminescence properties of QDs, resulting in color changes in the nanoprobes.^5^

On the other hand, smartphones have also developed rapidly as portable gadgets that can be carried in our pockets for communication, processing, computation, and even sensing applications. Smartphones primarily cover most people’s needs for a phone, digital camera, media player, GPS navigation, and cloud services by operating software applications (APPs). Smartphones have completely transformed our lives and even evolved into a potential and adaptable platform for scientific research.^6^

Smartphones are a new trend in analytical tools for biochemical detection due to their portability and widespread availability. Smartphones have been extensively integrated with fluorescence nanosensors,^7^ colorimetric nanosensors,^8^ electrochemical nanosensors,^9^ and microscopic bioimaging platforms.^10^ The smartphone colorimetric nanosensors were developed based on image capture with digital cameras and image processing algorithms with smartphone software applications. Due to their potential real-time applications in food safety,^11^ biomedicine,^12^ and environmental testing,^6^ colorimetric-based assays integrated with smartphones have received more and more attention.

Congo red is a hazardous azo dye derived from benzidine, which exhibits severe toxicity and is carcinogenic to both the environment and human beings. At the same time, Congo red is also highly stable in the environment.^13^ Using pigments and dyes in various industries produces vast quantities of Congo red-containing wastewater.^14^ However, compared to other azo dyes like methylene blue, methyl orange, and crystal violet, the detection of Congo red has yet to garner as much attention.^15^ Currently, the standard methods to detect Congo red are the electrochemical approach,^16^ spectrophotometry,^17^ fluorescence detection,^18^ and high-performance liquid chromatography (HPLC).^19^ The electrochemical approach was developed by measuring the cyclic voltammograms of Congo red degradation by graphene oxide nanoparticles produced on glassy carbon electrodes. Metal ions like barium chloride, ammonium sulfate, copper acetate, and potassium nitrate were observed as interference.^16^ The development of the spectrophotometric approach for Congo red detection was based on the catalytic activity of silver nanoparticles toward Congo red oxidation via potassium bromate using a hydrochloric acid solution. Foreign ions such as Cr(VI), Cu(II), and Fe(III) may interfere with this method. Besides, using hydrochloric acid to prepare the samples is also an unfriendly environmental approach.^17^ The fluorescence method provides high sensitivity and selectivity for industrial waste, bioimaging, and intracellular Congo red detection. However, environmental factors, instrumental bias, or excitation light sources easily influence the fluorescence detection methods, which may produce positive or negative false.^20^ The techniques mentioned above may consider labor-intensive costs, poor sensitivity, less selectivity, or longer operation times. In order to enhance environmental protection and minimize organic dye pollution, establishing an efficient Congo red monitoring platform is still of very high concern.

**Scheme 1:**
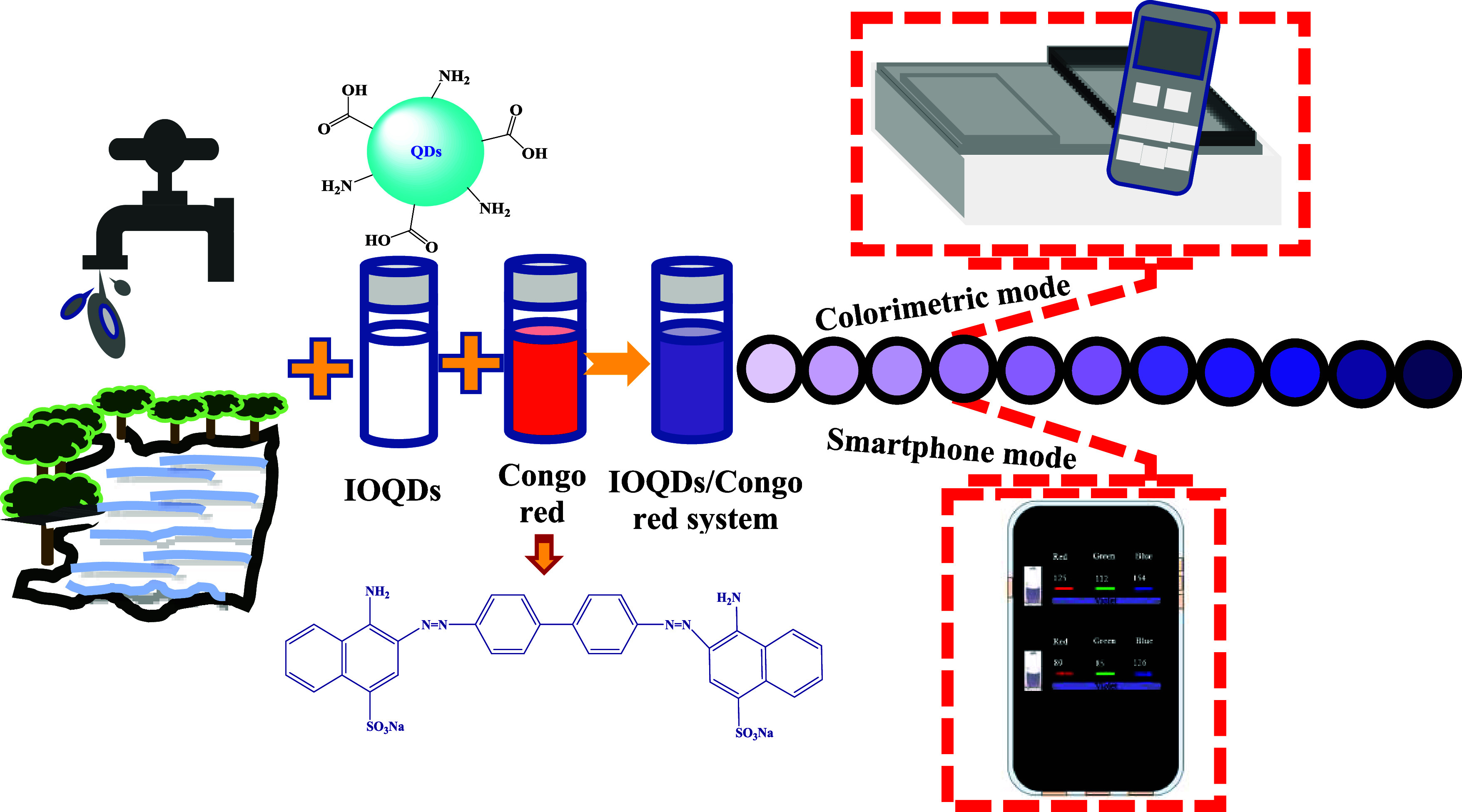
Illustration of Congo Red Detection Utilizing Dual Colorimetric and Smartphone-Assisted Mode

QDs offer better features than organic chromophores, such as higher fluorescence quantum yields, superior photostability, less toxicity, narrow and symmetric emission spectra, and broader absorption spectra.^21^ Therefore, QDs were widely applied in catalysts,^22^ biosensors,^23^ and environmental applications.^24^ Previously, our group prepared iron oxide quantum dots (IOQDs) using glutamic acid as a capping agent, and tetracycline in urine could be determined based on the fluorescence quenching phenomenon of IOQDs.^25^ The previous study found that the IOQD suspension could be formed under a high ionic strength or higher temperature conditions. Once the suspension is filtered, the supernatant IOQD solutions exhibit a quantum yield (QY) that is higher than that of the suspension form. Using these filtered IOQD solutions, the Congo red level in aqueous solutions can be visually detected using smartphone APPs or ultraviolet–visible (UV–vis) spectrometry (Scheme 1) with good selectivity, increasing its on-site real-time detection applicability. Even without a smartphone, naked-eye observation of Congo is also feasible using the sensing method developed in this study.

## Experimental Section

### Reagents and Solvents

NaI, dioxane, and quinine were obtained from Alfa Aesar. NaBr, CaCl_2_·2H_2_O, ZnCl_2_, KCl, NaCl, and NH_4_Cl were purchased from Showa Chemical. NaF was purchased from Across Organic. Ethanol was purchased from Echo. Tetrahydrofuran was obtained from Macron Fine Chemicals. Organic dyes, including Congo red, alizarin, allura red, brilliant blue, eosin, erythrocin, karmoisin, methyl orange, methyl red, purpurin, remasol red, rhodamine 6G, rhodamine B, rose Bengal, sunset yellow, and tetrazine, were purchased from Alfa Aesar. Dimethylformamide was obtained from JT Baker. Analytical grade solvents were used in all experiments, and all of the chemicals were used as they were received without further purification. A Millipore Simplicity Milli-Q ultrapure water system was used to prepare the aqueous solutions.

### Apparatus and Instruments

The apparatus used to prepare nanoparticles, quantum dots, and analytical solutions was a Pyrex glass. The fluorescence spectra of the IOQDs were measured with a Varian Cary Eclipse Fluorescence Spectrophotometer. The UV–vis absorption spectra were measured using a Spectra Academy SV-2100 UV–visible Spectrometer. The pH of the solutions was measured using a Hotec PH-10C pH meter. The identification of functional groups was performed by Fourier-transform infrared (FTIR) spectroscopy (ALPHA FTIR Spectrometer, Bruker). Particle size mensuration was performed by a Nanoplus HD-zeta/Nano Particle Analyzer. A Hitachi HT-7700 transmission electron microscope (TEM) with an accelerating voltage of 100 kV was used to examine the morphologies of the IOQDs. A Bruker Power XRD System (D2-Phaser) was employed to obtain the crystalline structure data of iron oxide NPs and IOQDs. A Quantum Design MPMS-XL7 Vibrating Sample Magnetometer System was implemented to measure the magnetic properties of the prepared IOQDs.

### Preparation of the Suspended IOQDs

Fe_3_O_4_ NPs and IOQDs were synthesized following our previous work^26^ with some modifications. In brief, 1.0 g of glutamic acid mixed with 10 mL of Fe_3_O_4_ NP solutions (1 mg/mL) was poured into a hydrothermal reactor tube and was introduced to a high-temperature oven at 200 °C for 14 h. Subsequently, the reactor was cooled to ambient temperature, and the solution was filtered using Whatman filter paper to remove the unreacted Fe_3_O_4_ NPs. It can be found that the suspended IOQDs appeared in 1 h under room temperature. After the suspended IOQDs appeared, the IOQD suspension solution was filtered again using a 0.22 μm membrane filter to remove the suspended IOQDs. The supernatant solution was collected and stored at 4 °C before further application.

### Stability Study of IOQDs

Different concentrations of NaCl were added to the solutions of IOQDs (final concentrations of 0–1000 mM) to study the ionic strength effect on the IOQDs. The pH effect of IOQDs was examined by adjusting the pH of IOQD solutions using concentrated aqueous HCl and NaOH aqueous solutions. The photostability of IOQDs was determined by exposing IOQDs to a white light LED and UV light (λ = 265 and 365 nm) for up to 30 min. The thermal stability of IOQDs was evaluated by heating them between 25 and 100 °C; the long-term stability of IOQDs under chilled temperature (5 °C) and room temperature was monitored for 30 days. Fluorescence spectra of the IOQDs were recorded to evaluate their stability using an excitation wavelength of 330 nm in each case.

### Colorimetric Sensor for Congo Red

Various organic dyes were used to evaluate the performance of the IOQDs as colorimetric sensors for Congo red. Each organic dye was mixed separately with the IOQD solutions with a fixed concentration of 50 μM. The color change was observed and measured after 10 min of incubation. Sensitivity and selectivity studies for Congo red were evaluated by observing the color change of the IOQDs.

### Smartphone Device Detection of Congo Red

A smartphone-based colorimetric detection device was designed to detect Congo red more accurately and precisely. The gadget’s components included a detecting cell and a smartphone (Figure 4a). A Mazepoly USB LED light (type YK-D06, China) with a 1.2 W power output was positioned at the top of the detecting cell, and the IOQDs/Congo Red mixed solution in a microcentrifuge tube was put on the spotted vial patch in the center of the detecting cell. A smartphone (iPhone 13) was used to acquire the images and convert them to RGB values using the Colorimeter X APP. The images were taken by holding the camera at a 45° angle before the detecting cell.

## Results and Discussion

### Characterization of the Prepared IOQDs

**Figure 1:**
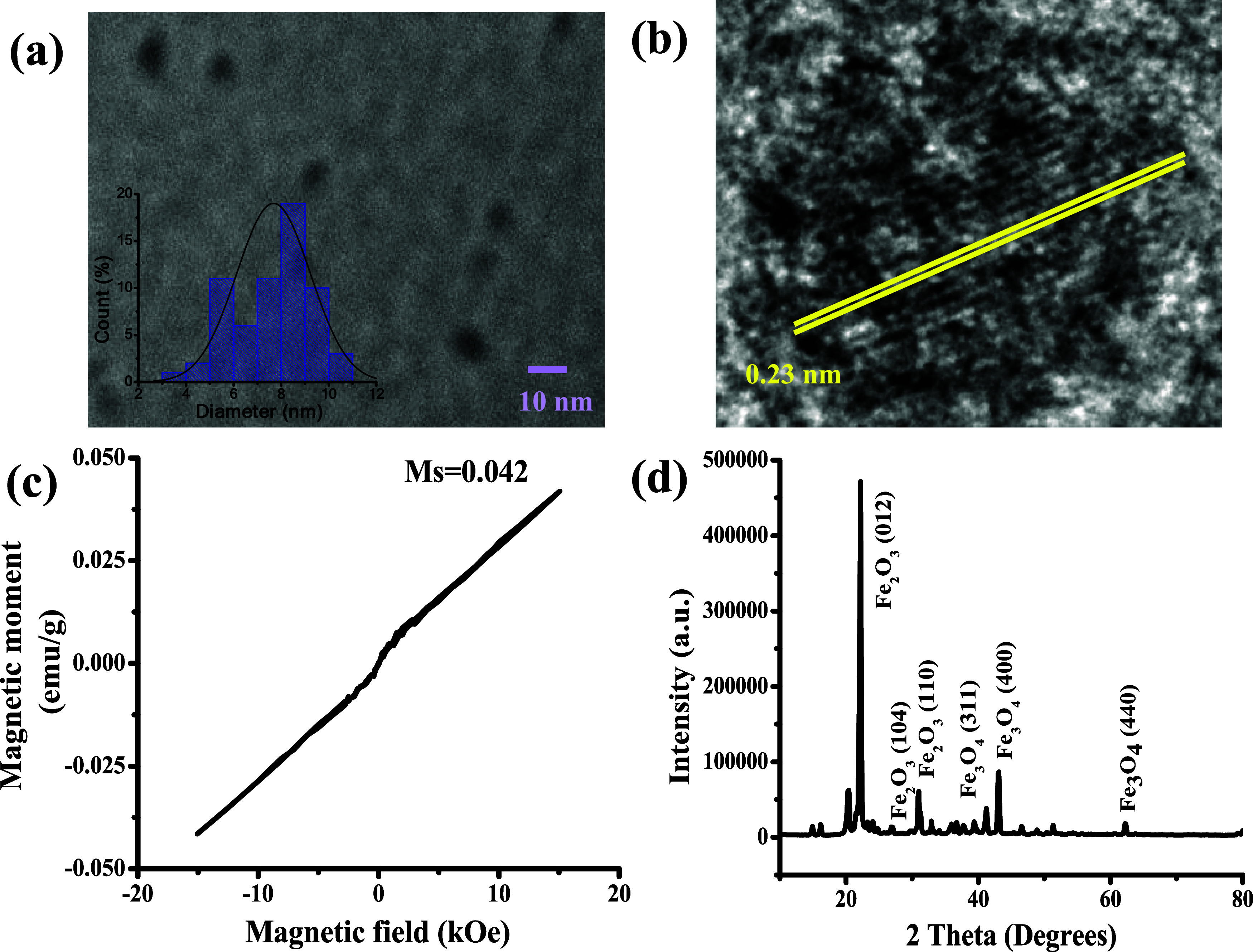
(a) TEM image (inset: particle size distribution), (b) HR-TEM image, (c) hysteresis loop, and (d) powder XRD spectrum of the suspended IOQDs synthesized in this study.

The TEM image (Figure 1a) demonstrates the surface morphology and particle size distribution of IOQDs prepared in this study. The filtered suspended IOQDs have average sizes of about 7.75 ± 0.19 nm and have a well-developed, uniform, and spherical-shaped morphology. Crystalline IOQDs’ (222) diffraction plane contributed to the lattice fringes observed in the high-resolution TEM (HR-TEM) image (Figure 1b) at a distance of 0.23 nm.^27^ The low magnetization saturation (Ms), as shown in Figure 1c, confirmed that IOQDs had limited magnetic properties, possibly due to the surface effect (i.e., oxidation).^28^ Powder X-ray diffraction (XRD) patterns (Figure 1d) were applied to determine the crystalline structures of the IOQDs. The observed diffraction peaks at 2θ of 22.4, 27.0, 30.9, 41.3, 43.0, and 62.2° were ascribed to the (012) plane of Fe_2_O_3_, (104) plane of Fe_2_O_3_, (110) plane of Fe_2_O_3_, (311) plane of Fe_3_O_4_, (400) plane of Fe_3_O_4_, and (440) plane of Fe_3_O_4_, respectively.^25^ It is noted that the broad diffraction pattern at 2θ of 21°, which is attributed to the graphitic planes of carbon materials,^29^ is not observed. Based on these results, the successful formation of IOQDs is confirmed.

### Optical Properties of IOQDs

**Figure 2:**
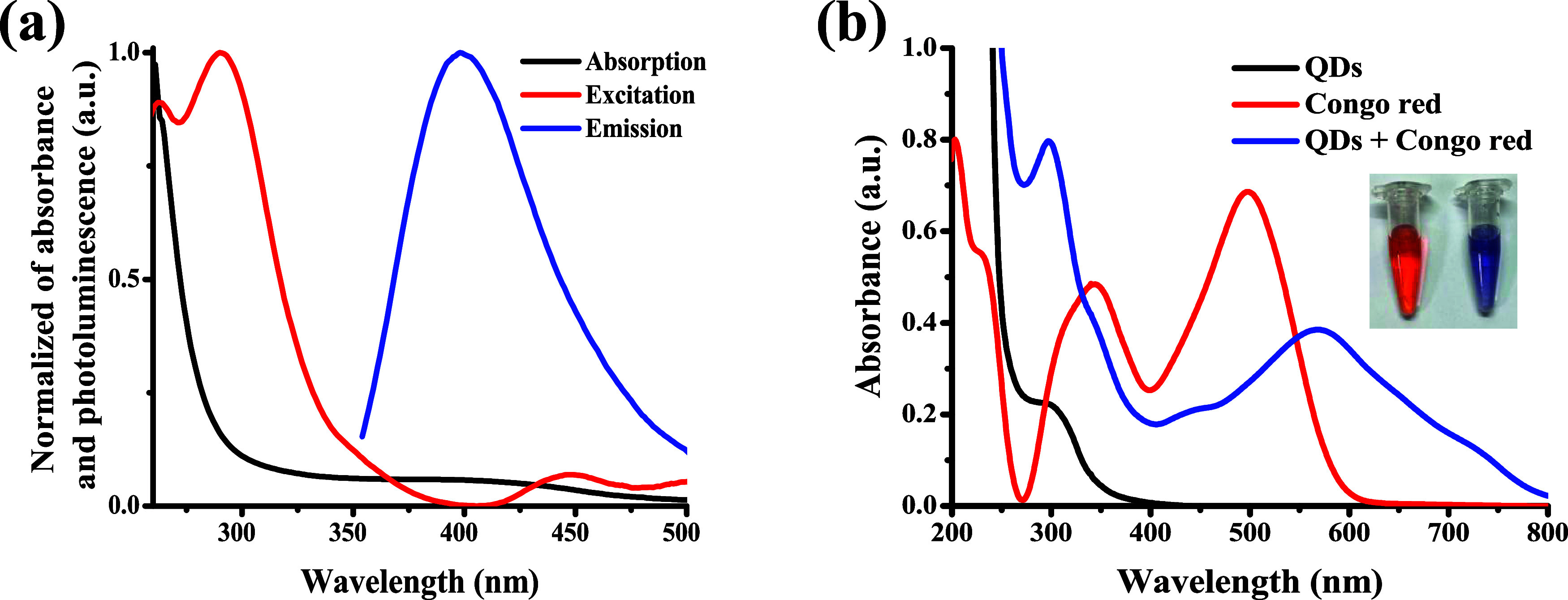
(a) Absorption, excitation, and emission spectra of the suspended IOQDs. (b) UV–vis spectra of IOQDs, Congo red, and IOQDs/Congo red system (inset: color changes of 50 μM Congo red before (left) and after (right) mixing with IOQDs).

UV–vis absorption, fluorescence excitation, and emission spectra of IOQDs synthesized in this study are shown in Figure 2a to study the optical properties of the suspended IOQDs. The IOQDs displayed an absorption band at 305 nm, and the maximum fluorescence emission appeared at 402 nm with an excitation wavelength of 330 nm. The fluorescence QY of the suspended IOQDs was calculated to be 13.80%, higher than that without suspension (Q.Y. = 9.93%).^30^

The fluorescence properties of IOQDs are directly related to their surface structure, morphology, and stability. The fluorescence characteristic of IOQDs was adopted to evaluate the stability of IOQDs under different conditions. Figure S1a shows the fluorescence intensity variation of IOQDs under LED light. As can be seen in the figure, the fluorescence intensity of IOQDs reduced by around 10% after 20 min of exposure and then remained steady. In contrast, the fluorescence intensity of IOQDs shows different behaviors under UV radiation. The fluorescence intensities of IOQDs decreased in the initial 5 min, and the fluorescence response returned after exposure to 10 min of UV radiation. Based on these results, the as-prepared IOQDs exhibited good stability and anti-photobleaching properties. In addition, the influence of ionic strength on the IOQDs’ fluorescence was also investigated (Figure S1b). As can be observed from the figure, the suspended IOQDs had no noticeable fluorescence change in the presence of sodium chloride, even under a concentration as high as 1000 mM. Notably, no further aggregation was observed under a high ionic strength, which can be concluded from the fact that the suspended IOQDs were electrostatically stable under high ionic concentrations.

The fluorescence intensities of IOQDs reduced as the temperature elevated due to the dissociation of passivants on the surface of IOQDs (Figure S2a).^31^ Interestingly, the IOQDs could return to the initial FL intensity after cooling to the ambient temperature (25 °C), suggesting that the surface of IOQDs would not be oxidized due to covalent bond protection.^32^ Furthermore, by adjusting the temperature from 25 to 100 °C, the reversible FL quenching and recovery of IOQDs was observed through four cycles, as shown in Figure S2b. The suspended IOQDs remained stable as the temperature was altered through several cycles, indicating the thermostability of IOQDs.

### Colorimetric Detection of Congo Red by IOQDs

**Figure 3:**
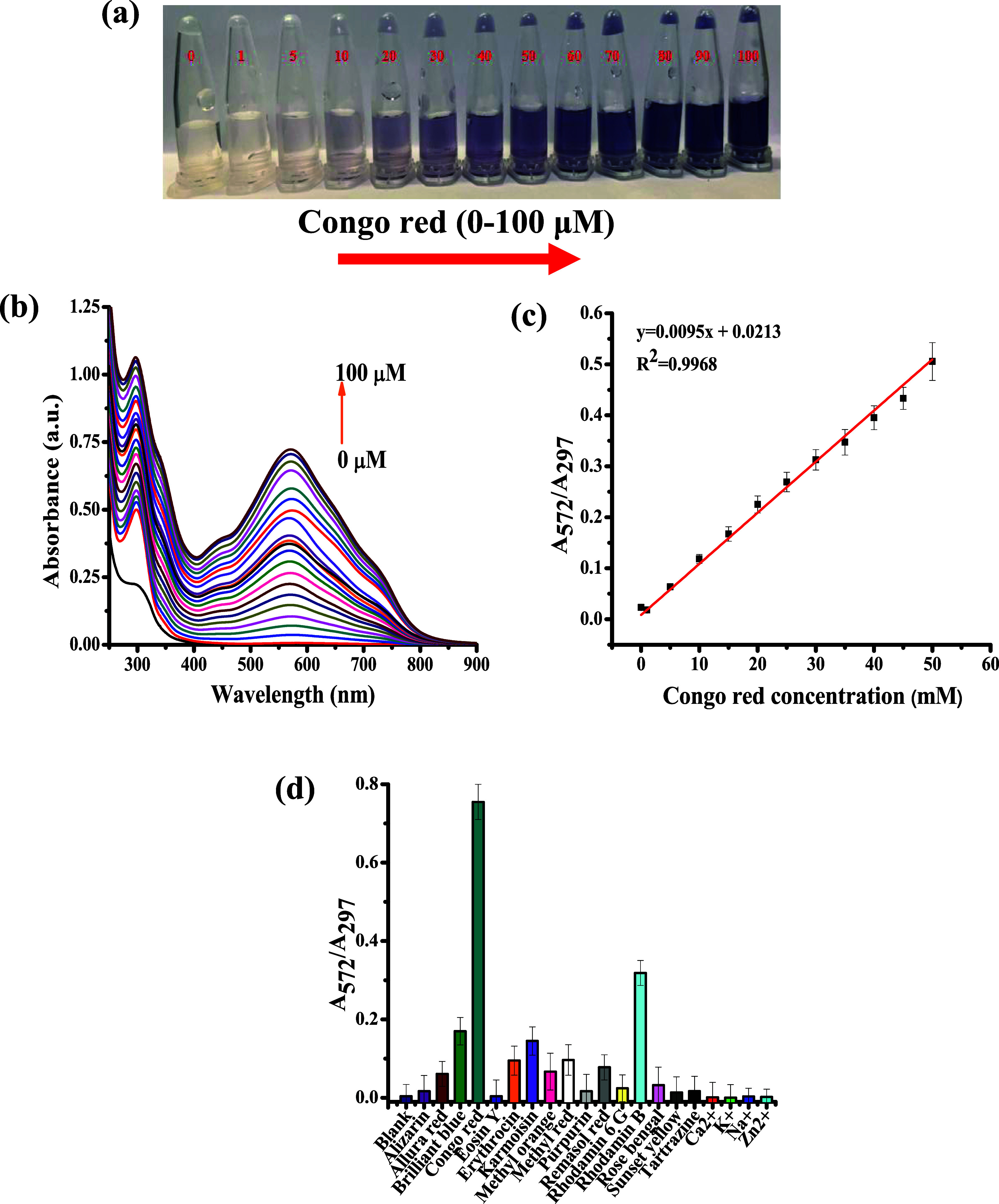
(a) Color changes of the IOQDs/Congo red system under different Congo red concentrations. (b) The UV–vis absorption spectra of the IOQDs/Congo red system under different Congo red concentrations. (c) The calibration curve of Congo red and the ratio values of absorbance at 572 nm to the absorbance at 297 nm (*A*_572_/*A*_297_). (d) The ratio values of *A*_572_/*A*_297_ of IOQDs in the presence of different dyes and metal ions, where the concentration of the examined species is 50 μM and the pH of all of the examined solutions is 3.0.

Figure 2b shows the UV–vis absorption spectra of IOQDs, Congo red, and Congo red mixed with IOQDs. As can be seen in the figure, IOQDs exhibited a main characteristic peak at 305 nm, which can be ascribed to the *n*–π* transition of the C=O bond on the IOQDs’ surface. The absorption spectrum of Congo red shows two main absorption bands at 340 and 496 nm, attributed to the π–π* transition of the aromatic ring and the *n*–π* transition of the lone pair present in the N atom of the azo moiety, respectively.^33^ After mixing Congo red with IOQDs, the characteristic band of IOQDs dramatically enhanced, and the *n*–π* transition in the N atom of the chromophoric −N=N– azo moiety of Congo red^34^ was red-shifted from 496 to 572 nm. The significant red shift suggests a strong interaction between the carboxyl groups of IOQDs and azo groups of Congo red. The absorbance at 572 nm increased as the concentration of Congo red increased. Similar behavior was observed at the absorbance peak of ∼297 nm (Figures 3b and 5b, IOQDs/Congo red system). The inset of Figure 2b exhibits the color of Congo red in deionized water (left) and that mixed with IOQD solutions (right). As can be seen in the figure, the color of Congo red solutions changes from red to blue right after mixing with IOQDs, which can be used for visual detection of Congo red.

The incubation time and pH values were optimized for rapid response and sensitive detection of Congo red. With different incubation times, the change in absorbance of the 50 μM IOQDs and Congo red mixture was investigated (Figure S3a), and the absorbance ratio values were stable right after mixing and remained unchanged for up to 30 min. The images of the IOQDs/Congo red (50 μM) sensing system under different pH are displayed in Figure S3b. The IOQDs/Congo red color changed when the pH was 2 and 3. Meanwhile, the color of the remaining pHs did not change and suffered from aggregation. The fluorescence response and absorbance of the IOQDs to 50 μM Congo red are shown in Figure S3c,d. pH 3 was selected as the optimal pH.

### Sensitivity and Selectivity of the Suspended IOQDs

Under those previously mentioned optimized experimental conditions, the capability of IOQDs to detect Congo red was examined by measuring the change in the UV–vis absorption spectra and RGB value of the probe under different Congo red concentrations. The colorimetric mode of IOQDs without and with Congo red exhibited colorless and violet colors, respectively, as demonstrated in Figure 3a. Upon gradually increasing Congo red concentrations from 2 to 100 μM, the IOQDs probe’s color steadily changed, and the absorbance at 297 and 572 nm increased continuously (Figure 3b).

Figure 3c demonstrates a linear increase in the absorbance ratio values of 572 to 297 nm (*A*_572_/*A*_297_). The linear fitting in the range of 2–50 M provided a correlation coefficient of 0.9968, and the detection limit was calculated as 0.89 μM by a method of the signal-to-noise ratio of 3 (*S*/*N* = 3). The selectivity of IOQDs was examined using colorimetric mode in the presence of a variety of acidic organic dyes (methyl orange, Congo red, tartrazine, rose bengal, alizarin, eosin, allura red, sunset yellow, and brilliant blue) and different metal ions K^+^, Na^+^, Zn^2+^, and Ca^2+^ (Figure 3d). It was found that only Congo red had a noticeable absorbance ratio value change from 572 to 297 nm (*A*_572_/*A*_297_) in the presence of IOQDs.

The high selectivity of IOQDs for Congo red can be attributed to the spectral selectivity of UV−vis spectroscopy and the relatively strong interaction between IOQDs and Congo red. As for the spectral selectivity, even though several organic dyes showed UV−vis absorption band shifts after mixing with IOQDs, only Congo red showed significant absorption at 572 nm (Figure S4a,b). As for the relatively strong interaction between IOQDs and Congo red, the interaction between the surface functional groups of IOQDs and the amine group of Congo red (Figure 5a) caused the color change into violet color, as depicted in Figure 2b. These findings revealed that the IOQDs could be used as a colorimetric sensor for the selective detection of Congo red, even in the presence of other red dyes.

### Colorimetric Detection for Congo Red in Real Samples

To further examine the practical application potential of the prepared IOQDs, recovery experiments were performed to detect Congo red by evaluating the recoveries of Congo red in real samples (i.e., tap water and lake water samples) spiking with different concentrations of Congo red. Relative standard deviations (RSDs) of less than 5% and the recoveries between 91.52 ± 0.01 and 118.08 ± 0.01% were obtained, and the results are tabulated as Table 1, indicating the great potential of IOQDs for the detection of Congo red in actual samples.

**Table 1:** Colorimetric Detection of Congo Red in Real Samples Using IOQDs

sample	spiked concentration (μM)	Congo red found (μM)	recovery (%)	RSD (%)
tap water	0	ND		
	10	11.33 ± 0.30	113.25 ± 0.03	2.65
	30	32.71 ± 0.94	109.02 ± 0.03	2.88
	50	47.54 ± 0.82	95.09 ± 0.02	1.73
lake water	0	ND		
	10	11.81 ± 0.07	118.08 ± 0.01	0.61
	30	31.47 ± 0.70	104.90 ± 0.02	2.24
	50	45.76 ± 0.34	91.52 ± 0.01	0.75

### Smartphone-Based on-Site Congo Red Detection

**Figure 4:**
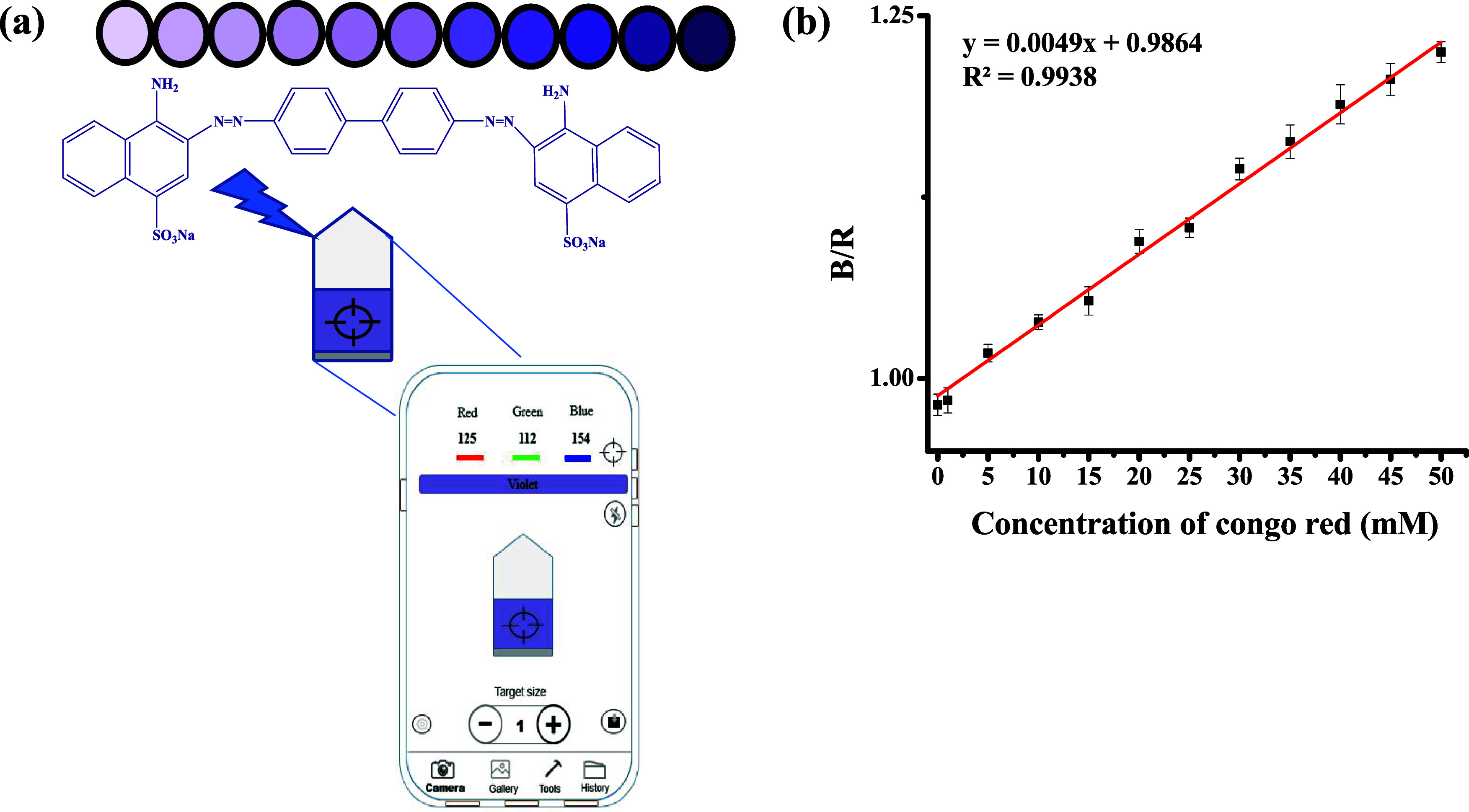
(a) Schematic illustration of the visual detection of Congo red using a smartphone. (b) The concentration relationship between the intensity ratio of the blue and red channels (B/R) and Congo red ranges from 0 to 50 μM. The pH of all of the examined solutions is 3.0.

To extend the applicability of the IOQDs for detecting Congo red, i.e., the on-site in situ examination, a smartphone with the Colorimeter X APP installed is used to analyze the image captured on-site (Figure 4a). For in situ quantitative analysis, the Colorimeter X APP can transform image color data into digital values that represent the red (R), green (G), and blue (B) color channels.^35^ Each color has a density range value from 0 to 255. Changes in color in a small range of RGB values can be identified and counted. As the chromogenicity of IOQDs/Congo red increased, the B/R ratio value gradually increased due to the increasing concentration of Congo red. As shown in Figure 4b, the ratio of the blue (B) to red (R) channel (B/R) is linearly dependent on the Congo red concentration in the range from 1 to 50 μM. The detection limit by using the smartphone-based method is calculated to be 0.58 μM. Table 2 provides a comparison of the detection results for Congo red. As can be found in the table, the development of the smartphone-based Congo red detection method in this study offers benefits such as low cost, straightforward operation, and signal readout; therefore, the color imaging-based approach may present a suitable platform for the quick, precise, and on-site measurement of Congo red.

**Table 2:** Comparison of Different Methods for Congo Red Detection

method	material	detection time	dynamic range	LOD	references
electrochemical	graphene oxide-modified electrode	100 s	0.01–0.2 μM	0.24 μM	(16)
electro-oxidation	glassy carbon electrode		N/A	0.10 μM	(36)
spectrophotometric	nanosilver	150 s	1.1–345 μM	0.60 μM	(37)
HPLC		7 min	0.04–1.4 μM	0.01 μM	(19)
HPLC	molecularly imprinted polymers	60 min	0.01–1.4 μM	0.07 μg kg^–1^	(38)
electrochemical	SWVatAuNPs/CPE	15 min	1–30 and 50–200 μM	0.07 and 0.70 μM	(39)
fluorescence	N-CDs	2 min	0–10	0.035 μM	(40)
fluorescence	Ca, N, S-CDs	1 min	0.2–1.2	0.058 μM	(41)
colorimetric	iron oxide quantum dots	2 min	1–50 μM	0.89 μM	this work
smartphone-assisted detection	iron oxide quantum dots	2 min	1–50 μM	0.58 μM	this work

To assess the feasibility of the smartphone-based sensing platform, tap water and lake water samples are examined using the developed methods. The treated samples were placed into the sample stage of the cell under an LED lamp, where the image of the sample cells was taken, and the smartphone analyzed the results. Table 3 lists the determination results using the smartphone-assisted method for detecting Congo red in real samples. The obtained recoveries were in the range of 91.52 ± 0.01–118.08 ± 0.01%, with relative standard deviations (RSDs) not exceeding 5%. High recoveries and low RSDs demonstrated that the smartphone-based detection of Congo red in real samples is highly feasible.

**Table 3:** Determination Results of Congo Red Detection Using Smartphone-Based RGB Analysis

sample	spiked concentration (μM)	Congo red found (μM)	recovery (%)	RSD (%)
tap water	0	ND		
	10	10.65 ± 0.26	106.45 ± 2.55	2.39
	30	31.07 ± 0.80	103.57 ± 2.57	2.57
	50	52.69 ± 1.57	105.38 ± 3.14	2.98
lake water	0	ND		
	10	11.81 ± 0.07	118.08 ± 0.01	0.63
	30	31.47 ± 0.70	104.90 ± 0.02	3.47
	50	45.76 ± 0.34	91.52 ± 0.01	2.06

### Feasible Mechanism for Congo Red Detection

**Figure 5:**
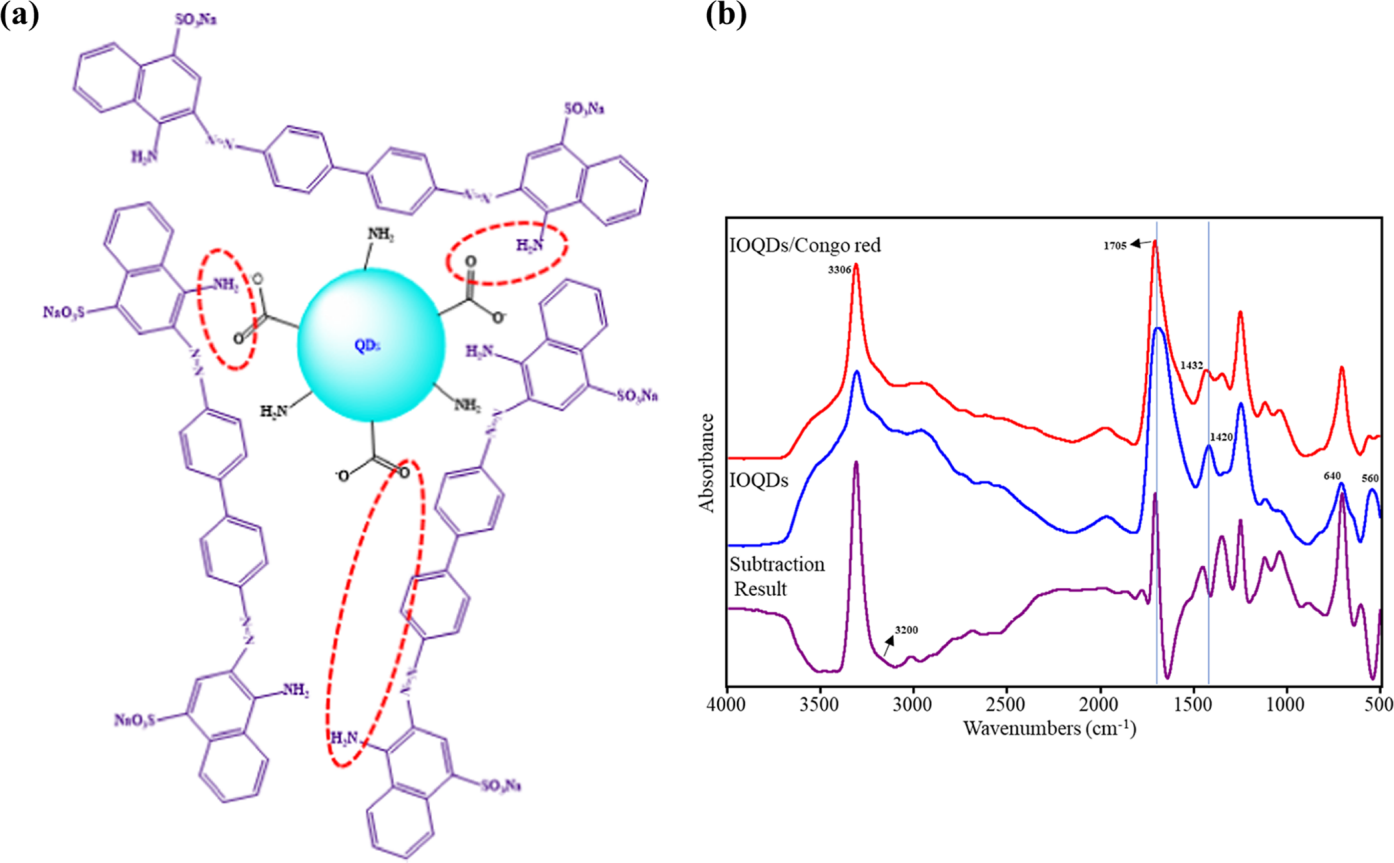
(a) Feasible mechanism of IOQDs for Congo red detection. (b) FTIR spectra of IOQDs, the IOQDs/Congo red system, and the subtracted result.

The feasible sensing mechanism for colorimetric Congo red determination is proposed in Figure 5a. As earlier reported, it is assumed that a relatively strong interaction between the carboxyl group on IOQDs’ surface and the amine group of Congo red resulted in a bathochromic shift.^42^ IOQDs catalyzed the chromogenic reaction of Congo red to form an IOQDs/Congo red system with strong absorption at 297 and 572 nm. A strong interaction between IOQDs and Congo red shifted spectral bands, as shown in Figure 2b. IOQDs exhibited a characteristic peak at around 305 nm, and there are two characteristic peaks at around 340 and 496 nm in the spectra of Congo red. The absorbance at 572 nm increased as the concentration of Congo red in the IOQD solution increased, whereas the absorption band at 297 nm also increased (Figure 3b, IOQDs/Congo red system). Therefore, the color change of the solution can be revealed due to the change in the absorption of two wavelengths (297 and 572 nm). Figure S3b illustrates our findings, which reveal that the violet hue only emerges at pH values of 2 and 3, with precipitation and the natural Congo red color occurring at pH values of 4–10. When the pH is below 5, the Congo red structure is cationic, and when it is above 5, it is anionic, giving rise to blue and red hues, respectively. Conversely, at pH of 2 and 3, the –COO– and OH– groups on IOQDs’ surface interact with the azo group of Congo red to produce a violet color; at other pHs, either an acidic or basic conditions did not alter the color of Congo red. Meanwhile, other tested dyes examined at a pH of 3 did not show any color change in the presence of IOQDs. To further investigate the mechanism of the IOQDs/Congo red sensing system, the FTIR spectra of IOQDs and IOQDs/Congo red were measured and compared (Figure 5b). Fe–O stretching and Fe–O bending vibrations of the IOQDs at 560 and 640 cm^–1^ are found. The other two peaks centered at 1697 and 1420 cm^–1^ corresponded to the antisymmetric carboxylate group stretching and the distal α-carboxylic groups on IOQDs. –NH_2_ asymmetric stretching at 1620 cm^–1^ and symmetric stretching at 1520 cm^–1^ were also observed, indicating the presence of amine groups on IOQDs’ surface. In comparison, IOQDs/Congo red infrared absorption spectra showed that the vibrations of −OH stretching were slightly red-shifted due to hydrogen bondings. Besides, due to hydrogen bonding, the absorption band of –NH centered at around 3306 cm^–1^ sharpened, possibly caused by the unitary intermolecular interactions between IOQDs and Congo red molecules. The carboxyl group exhibits hydrogen bonding and shifted^43^ to 1705 and 1432 cm^–1^. To enhance the visibility of the intermolecular interaction between IOQDs and Congo red, the spectral subtraction result of IOQDs/Congo red mixtures to IOQDs is also plotted in Figure 5b. As can be seen in the subtraction result, an increase in absorption at ∼3200 cm^–1^, accompanied by a decrease in absorption at 3430 cm^–1^, suggests the presence of hydrogen bondings.^44^ A first-order derivative spectra-like result between 1706 and 1643 cm^–1^ can also be found, providing more direct evidence of hydrogen bondings. Significant vibrational band shifts of Congo red can be observed in the spectral subtraction results compared to those without mixing with IOQDs.^45^ For example, the –N=N– stretching vibration of Congo red shifted to 1432 cm^–1^ from 1446 cm^–1^, the C–N bending vibration of Congo red shifted to 1347 cm^–1^ from 1360 cm^–1^, and the S=O stretching vibration of sulfonic acid on Congo red shifted to 1036 cm^–1^ from 1062 cm^–1^. All these findings suggested the significant interactions between Congo red and IOQDs, making the visible detection of Congo red possible.

## Conclusions

In this study, we have developed a convenient, fast, and simple sensing platform for detecting Congo red based on the dual mode of colorimetric and smartphone devices. The developed IOQDs can be applied as a colorimetric probe for detecting Congo red with a dynamic range between 1 and 50 μM, and the detection limit was calculated to be 0.89 μM. To further increase the possibility of the on-site direct detection of Congo red, a smartphone installed with a proper APP was introduced to analyze the RGB values of the captured images, which can predict the concentration of Congo red in real samples. The smartphone’s dynamic range was from 1 to 50 μM with a detection limit of 0.58 μM. On the other hand, both methods successfully detected Congo red in real samples, such as lake water and tap water samples. In conclusion, this method offered on-site real-time detection, a more comprehensive linear range, selectivity, low cost, and easy detection, which make it a highly complementary detection method for Congo red.

## Supporting Information Available

Details on the stability study of IOQDs; selectivity trial results of IOQDs; and the real-time video of IOQDs mixed with Congo red are available via the internet (PDF)
Real-time video of IOQDs mixed with Congo red are available via the internet (AVI)

## Supplementary Material

ao4c08644_si_001.pdf

ao4c08644_si_002.avi
