# Supplementary material for: Colorimetric and Smartphone-Based Dual-Mode Rapid Detection of Congo Red Using Iron Oxide Quantum Dots
Source: ACS Omega. 2024 Nov 7;9(46):46600–9. doi: 10.1021/acsomega.4c08644 (PMC11579718; doi:10.1021/acsomega.4c08644)
Supplement: Supplementary file 1 — ao4c08644_si_001.pdf [file ao4c08644_si_001.pdf]

Supporting Information

for

Colorimetric and Smartphone-based Dual-mode Rapid Detection of Congo Red Using Iron Oxide Quantum Dots

Sri Sudewi,[1] Chien-Hung Li,[2] Venkata Sai Sashankh Penki,[2] Muhammad Zulfajri,[3] Naorem Jemes Meitei,[2] and Genin Gary Huang,[2,4,5,*]

[1]Department of Pharmacy, Faculty of Mathematics and Natural Science, Universitas Sam Ratulangi, Manado 95115, Indonesia
[2]Department of Medicinal and Applied Chemistry, Kaohsiung Medical University, Kaohsiung 80708, Taiwan
[3]Department of Chemistry Education, Universitas Serambi Mekkah, Banda Aceh, Aceh 23245, Indonesia
[4]Department of Medical Research, Kaohsiung Medical University Hospital, Kaohsiung 80708, Taiwan
[5]Department of Chemistry, National Sun Yat-sen University, Kaohsiung 80424, Taiwan

*To whom correspondence should be addressed.
E-mail: genin@kmu.edu.tw
Phone: +886-7-3121101#2810
Fax: +886-7-3125339

Figure S1

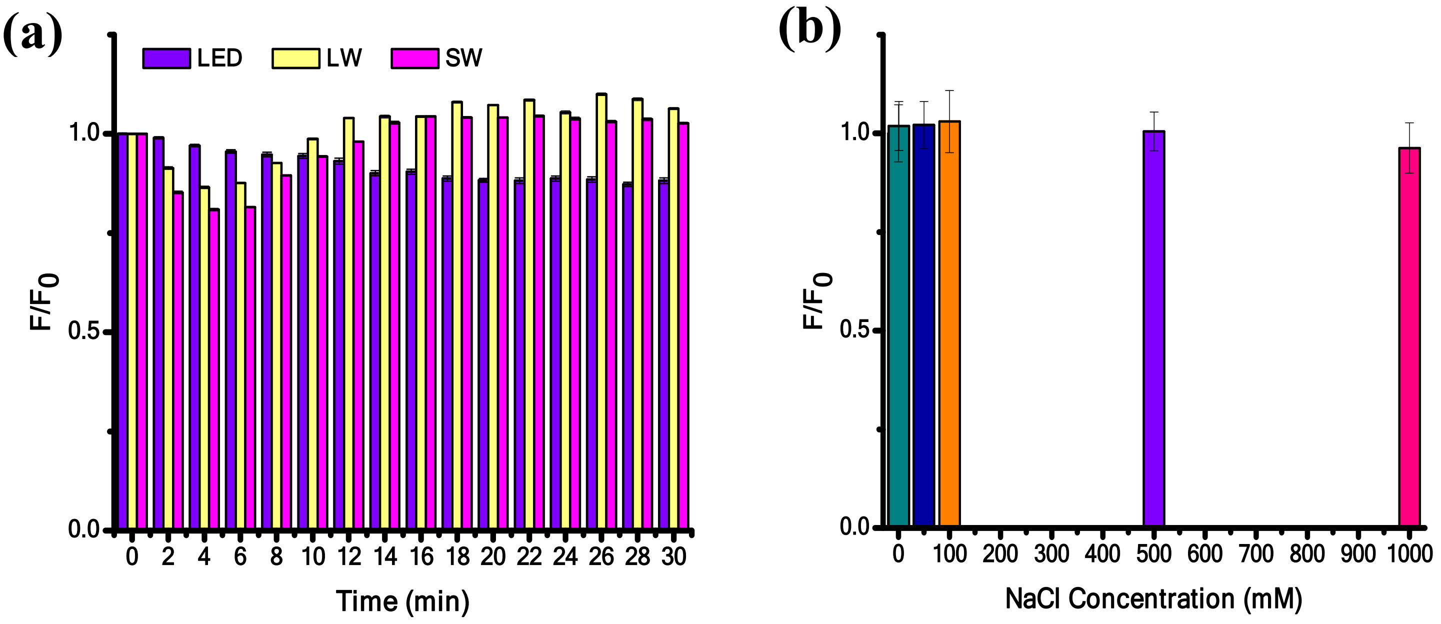

Figure S1. (a) Photostability of the IOQDs synthesized in this study. (b) Ionic strength effect on IOQDs.

Figure S2

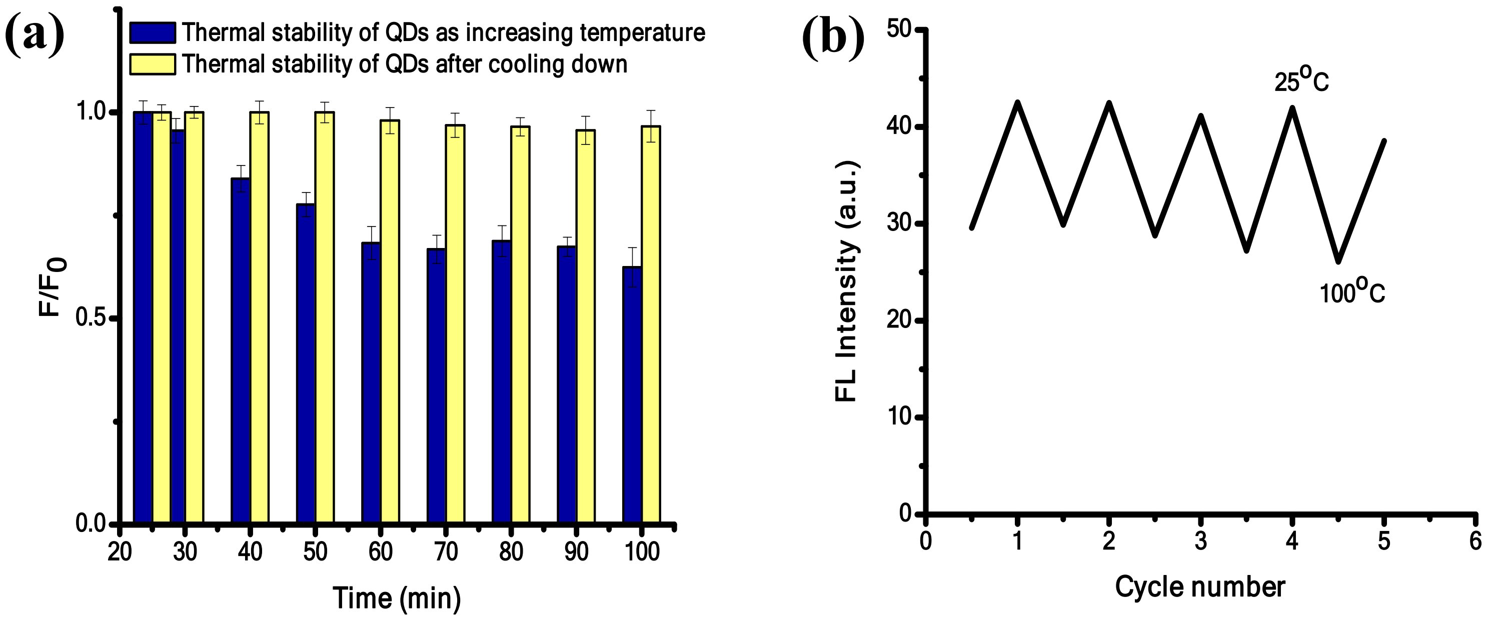

Figure S2. (a) Thermal stability of IOQDs during heating and after cooling down. (b) reversible fluorescence behavior upon cycling temperature between 25°C and 100°C of IOQDs.

Figure S3

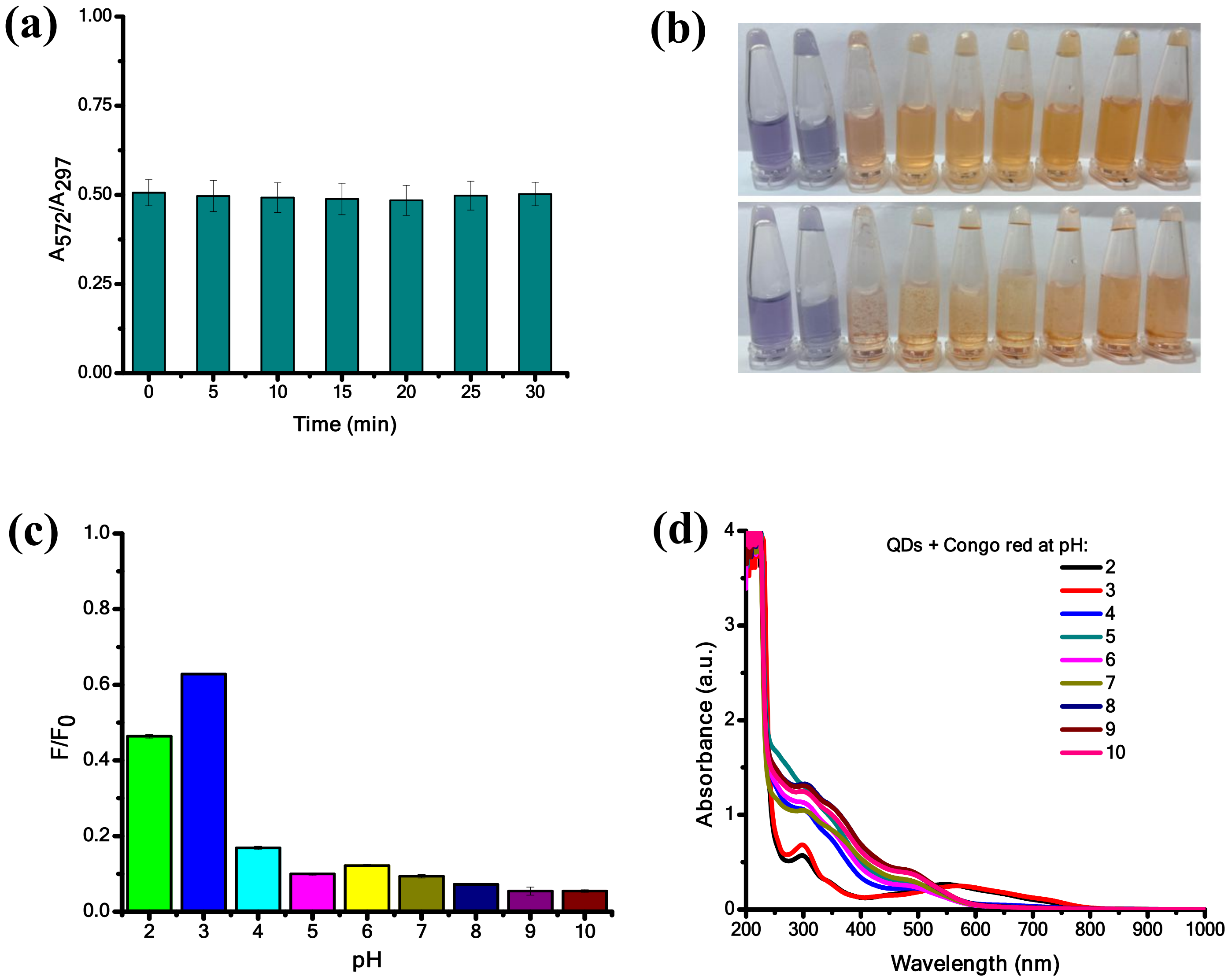

Figure S3. (a) Response time of IOQDs/Congo red system. (b) Images of IOQDs after the addition of Congo red under different pHs. (top: real time color change right after mixing with Congo red; bottom: observation after 5 min.) (c) relative fluorescence intensities ratio (F/F_0) of IOQDs/Congo red at various pHs. (d) UV-Vis absorption spectra of IOQDs/Congo red under different pHs. The concentration of Congo red is 50 μM and the pH of all the examined solutions is 3.0.

Figure S4

(a)

(b)

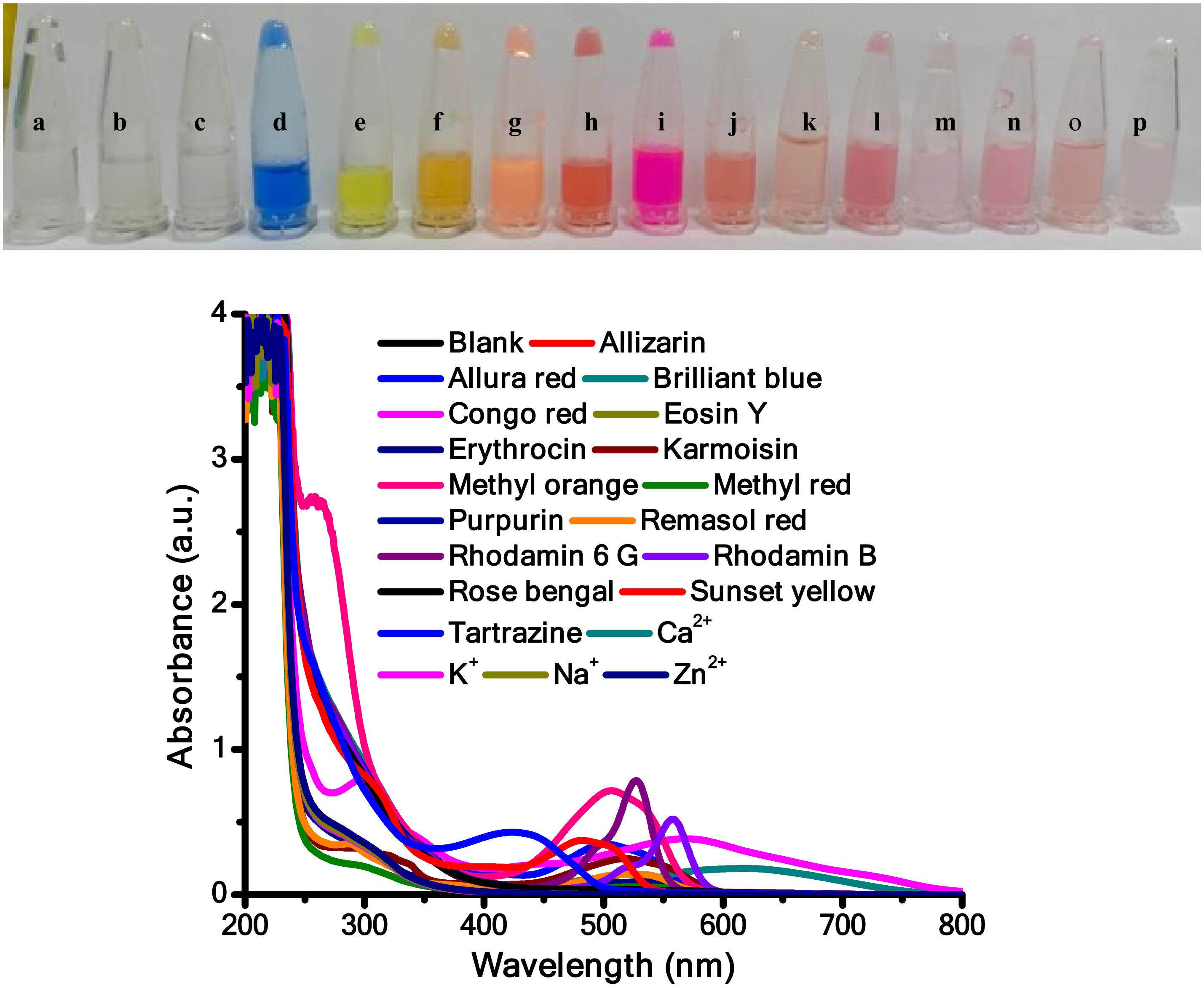

Figure S4. (a) Color of IOQDs in the presence of various organic dyes at the concentration of 50 µM (a: Blank/IOQDs+H_2O, b: Allizarin, c: Allura red, d: Brilliant blue, e: Eosin Y, f: Erythrocin, g: Karmoisin, h: Methyl orange, i: Methyl red, j: Purpurin, k: Remasol red, l: Rhodamin 6 G, m: Rhodamin B, n: Rose Bengal, o: Sunset yellow, p: Tartrazine). (b) UV-Vis absorption spectra of IOQDs in the presence of various organic dyes and metal ions where the concentration of all the examined organic dyes and metal ions is 50 µM and the pH of all the examined solutions is 3.0.
